# Found in translation: Integrating laboratory and clinical oncology research

**DOI:** 10.2349/biij.4.3.e47

**Published:** 2008-07-01

**Authors:** H Wagner

**Affiliations:** Division of Radiation Oncology, Penn State Hershey Cancer Institute, Hershey, Pennsylvania, United States

**Keywords:** translational research, clinical trials, targeted therapy, protons, radiation therapy

## Abstract

Translational research in medicine aims to inform the clinic and the laboratory with the results of each other’s work, and to bring promising and validated new therapies into clinical application. While laudable in intent, this is complicated in practice and the current state of translational research in cancer shows both striking success stories and examples of the numerous potential obstacles as well as opportunities for delays and errors in translation. This paper reviews the premises, promises, and problems of translational research with a focus on radiation oncology and suggests opportunities for improvements in future research design.

"A good many times I have been present at gatherings of people who, by the standards of the traditional culture, are thought highly educated and who have with considerable gusto been expressing their incredulity of scientists. Once or twice I have been provoked and have asked the company how many of them could describe the Second Law of Thermodynamics. The response was cold: it was also negative. Yet I was asking something which is the scientific equivalent of: Have you read a work of Shakespeare's?

I now believe that if I had asked an even simpler question -- such as, What do you mean by mass, or acceleration, which is the scientific equivalent of saying, Can you read? -- not more than one in ten of the highly educated would have felt that I was speaking the same language. So the great edifice of modern physics goes up, and the majority of the cleverest people in the western world have about as much insight into it as their neolithic ancestors would have had."

– CP Snow The Two Cultures (1959)

“Traddutore, traditore”

– Italian Proverb, loosely translated as

‘To translate is to betray.”

‘Translation is the action of interpretation of the meaning of a text, and subsequent production of an equivalent text, also called a translation, that communicates the same message in another language.’

– Wikipedia accessed 1 September 2008.

## INTRODUCTION

Twenty-five years ago when I was completing my Fellowship in Radiation Oncology there was little use of the term ‘translational research’, in cancer or elsewhere in medicine. There were clinicians and there were laboratory scientists. They kept different hours (6AM to 6PM for the clinicians, noon to midnight for the basic scientists). They tended to differ in age, dress, style of music, and a host of other superficialities. To some degree they represented two tribes within the common culture of science. While not yet speaking the different languages that Snow cites, they were clearly using quite different dialects.

But even then some saw the need to bring these two groups together in the service of a goal more exciting and more important what either could do alone. One of my first mentors combined a full time cardiology practice with running an immunology lab, which resulted in the development of diagnostic and therapeutic antibodies against cardiac glycosides [1]. During my Residency in Radiation Oncology at Tufts-New England Medical Center Hospital, we had a tradition that Radiation Physicists and Radiation Biologists would routinely attend our daily new patient presentation conference. Their questions, often unencumbered by the then current clinical dogmas, sometimes seemed to come out of left field, but perhaps that is where the best answers are to be found.

Translational research has now become somewhat of a buzzword in medicine, along with such concepts as ‘molecular medicine’ and ‘personalised medicine’. This is all to the good of medicine. The close intermingling of theory and practice, *in vitro* and animal models with the human reality of clinical practice, has brought significant advances both in our basic understanding of the biology of human cancer and in its treatment. Yet general acceptance of the concept does not mean that its implementation is either widespread or easy. And contrary to the simplistic “Bench to Bedside” slogan that we use to drum up support for translational research, in practice it is a complex dance from bench to bedside and back again, repeatedly, with not only the clinician and basic scientist as dancers but with the active (for good or bad) involvement of academic, governmental, and commercial onlookers and regulators. Recently the United States National Cancer Institute has created a Translational Research Working Group (TRWG) specifically charged with defining and improving the processes by which new compounds and/or devices are brought from initial conception or discovery to the point of clinical evaluation in patients [2]. The general overview of this process, as well as specific considerations apropos to the development of drugs for medical oncology and devices for radiation oncology, have been recently published [3-5]. In this article I will review some of the premises, explicit and implicit, that underlie the concept of translational research, give some examples of successful and unsuccessful attempts at their implementation, and address structural impediments and possible solutions. The high cost and relative inefficiency of the development of new treatments in oncology, with only a small portion of promising new therapies proving superior to established ones, mandates a review of our clinical research practices [6-8]. If the focus seems at times negative, this is not because I think that translational research is a failure, which I emphatically do not, but because I think that we often learn more from an analysis of our failures than a celebration of our successes.

### Principles of translational research

A core premise of translational research, whether in oncology or other disciplines, is that clinical trials are more likely to advance knowledge and lead to improved treatment when they are done in close collaboration with basic science. Trials should not simply compare clinical endpoints, such as survival, for patients given ‘Treatment A vs. Treatment B’ but also provide information which will allow testing of the proposed underlying mechanisms behind the two treatments. This will typically entail inclusion of some, if not all, of the following features:

- Design of clinical trials is based on laboratory observations regarding tumour biology and agents which may be able to perturb it.
- Intermediate endpoints which may address the clinical hypothesis being tested whether or not the primary endpoint is satisfied.
- Collection of data on additional endpoints (e.g. quality of life, economic impact on patients and caregivers).
- Collection of biologic specimens (tumour, normal tissue, blood, urine) for studies correlating DNA, RNA, protein, and drug metabolite information with clinical outcomes in this trial and hypothesis generation for later studies.
- Feedback of outcome data (e.g. local control, survival, patterns of failure) to basic scientists for evaluation of original study hypothesis and generation of new hypotheses and agents with which to test them.

In practice these criteria are fully met in only a minority of trials. Limitations in our understanding or the mechanisms of actions of agents, particularly ‘off-target’ effects, cost and difficulty of obtaining tissue specimens from patients, especially those with solid tumours – with which to make the desired correlations between administered agent, molecular effects in target tissues, and clinical outcomes, as well as commercial and academic pressures to succeed quickly – have all distorted the idealised research process. As it is currently practised, translational research is often too slow and ineffective in developing successful new therapies [9-10].

### Some Examples

The development of agents which can interfere with signaling pathways mediated through the epidermoid growth factor receptor (EGFR) signal pathway provides a good example of the translational research approach, including its successes, failures, and surprises. Over the past three decades, the existence of this pathway has been recognised , leading to description of its significance to tumour growth and frequent correlation of its activity with resistance of tumours to treatment such as radiotherapy, the development of several classes of agents including monoclonal antibodies directed against the receptor (e.g. Cetuximab) and low molecular compounds (TKI) which target the ATP binding site in the intramolecular kinase domain of the receptor , and the completion of clinical trials evaluating several of these either as single agents or in combination with radiation therapy or chemotherapy [11-15]. The result of this effort has been both the approval of several agents for clinical use and marked increase in our understanding of the biology of this signaling pathway and its perturbation in a variety of tumours. The observation that some groups of patients (such as nonsmoking Asian women with adenocarcinoma of the lung) had very high response rates to TKI led to the discovery of activating mutations in the kinase domain of the EGFR and a much better appreciation of the molecular heterogeneity of the family of diseases we call lung cancer. While this has been a translational success story in many respects, key elements have been frustratingly incomplete or absent. Despite the early interest in EGFR inhibition as a strategy for radiosensitisation, only one Phase III trial with Cetuximab in head and neck cancer has been completed and published. Several small Phase II trials with Cetuximab and chemoradiation have been reported in non-small cell lung cancer (NSCLC) and have led to a currently active RTOG Phase III trial. Correlation between either overexpression or mutation of the EGFR in subjects on these trials has been largely lacking. Even in the much larger trials looking at either Cetuximab or the TKI Gefitinib or Erlotinib in NSCLC, the lack of assay standardisation has left us with a somewhat confusing picture regarding the relative importance of mutations (which seek key for single agent activity of the TKI in NSCLC) versus overexpression of EGFR which may be sufficient for radiosensitisation. The relative lack of clinical success of the TKI as radiosensitisers is surprising in view of their *in vitro* activity, and it is only recently that we are beginning to recognise that while both antibodies such as Cetuximab and TKI will block EGFR mediated cell signaling, they do so via profoundly different ways with markedly different downstream effects. Such facets of EGFR activity as its nuclear localisation were unknown when these agents were developed, and the feedback from the clinic to the laboratory that has occurred with the results of first generation trials has greatly enhanced our understanding of EGFR biology.

### Inhibition of farnesyltransferase and ras

At about the same time that the EGFR story was developing, it was recognised that mutational activation of the ras protein was a major feature of a variety of malignancies. It was also observed that increased ras signaling was associated with radiation resistance in several cell lines [16]. As is often the case, this was first thought to be a simple phenomenon, and the importance of the various members of the ras protein family underappreciated. With the realisation that, for ras to play its role in cell signaling it required post-translational modification including attachment of a prenyl group to allow its incorporation into lipid membranes, the search was on for a selective inhibitor of the enzyme thought to be most responsible for this, ras farnesyltransferase (FTase). A number of compounds of varying structures were developed which were active FTase inhibitors and which showed impressive clinical activity against tumour lines harboring ras mutations.

In the clinic these compounds have been major disappointments, either as single agents or in combination with chemotherapy or radiation [17-18]. In hindsight, it seems that there were at least four key areas in which our basic understanding of this set of signaling pathways and its modification were inadequate to the task of developing active and specific therapeutic agents:

- Insufficient understanding of the complexity of the Ras isoform system in human malignancies.
- Incomplete understanding of alternate prenylation pathways (e.g. geranylgeranylation) when farnesyltransferase is inhibited.
- Other effects of the farnesyltransferase inhibitors. It is estimated that at least 20 other proteins contain the CAAX sequence targeting them for farnesylation. The effects of the agents developed as FTase inhibitors on these largely unknown targets is not known.
- Lack of clear understanding as to which pathways downstream of ras were critical for its effects on radiation sensitivity. Identification of such pathways might lead to more selective agents for altering radiosensitivity.

While the search for agents targeting the EGFR pathway and those targeting Ras began at about the same time, attracted considerable commercial interest, and were touted as heralds of the new molecular oncology, they have at present led to rather different endpoints. Detailed study of what went right and wrong in these two approaches may be valuable in improving future efforts at targeted drug development.

### Altered Radiation Fractionation in Head and Neck, and Lung Cancer

Prior to the development of chemical agents capable of altering the response of cells and tissues to radiation, it was recognised that alterations in radiation fractionation could differentially affect tissues. In classic experiments performed in the 1920s Regaud showed that small daily doses of radiation could produce sterility in rams while preserving skin, whereas unfractionated treatment caused desquamation without sterility [19]. This and similar observations led to the adoption of daily radiation fractionation for most malignancies. In the 1960s and 1970s data in model animal systems suggested that accelerating dose delivery, either by administering larger daily fractions (Accelerated fractionation) or keeping the fraction size small but giving two or more fractions on each treatment day (hyperfractionation), could increase local tumour control [20]. Among other possible benefits, such a technique could be widely adopted in radiation oncology centres throughout the world, required no new and potentially expensive technology, and did not require infusion of any sensitising or protecting drugs. Clinical trials were soon mounted in a variety of disease sites with particular interest being placed in ones such as tumours of the head and neck where control of locoregional disease is closely associated with both quality of life and survival. A recent meta-analysis has demonstrated that both the hyperfractionated and accelerated approaches have produced modest but significant improvements in local control, albeit with an increase in acute toxicity [21].

At about the same time that these trials were being implemented, other investigators were exploring the use of chemotherapeutic agents such as cisplatin and carboplatin as radiation sensitisers. Thus, by the time that results of the daily vs. accelerated or hyperfractionated radiation trials became mature, the baseline had changed and daily radiation as a single modality was no longer considered appropriate standard therapy for patients of good performance status. A somewhat similar situation arose a few years later in trials of daily radiation therapy with or without the anti-EGFR monoclonal antibody Cetuximab. This lack of coordination of trials resulted in the current situation in which we know that any of the three approaches, modified fractionation and dose escalation, concurrent chemotherapy with cisplatin, or concurrent biologic therapy with Cetuximab, is superior to conventional daily fractionation as a single modality but we do not know whether one of these approaches is ‘best’ for head and neck cancer patients in general or for specific patients.

It is unfortunate that we lack good correlative biologic studies to help individualise the choice of sensitiser in specific patients (or to identify those patients whose tumours would be controlled with conventional therapy and who could be spared the additional toxicities associated with more aggressive treatment). Several candidates have been suggested and appeared promising in small studies, such as proliferative index or potential doubling time for fractionation, ERCC1 expression for cisplatin, and EFRG expression for Cetuximab. Unfortunately it has not been possible, to date, to do these assays prospectively across different trials (some academic, some industry sponsored), to obtain a better ability to select the appropriate fractionation and sensitisation strategy on an individual patient basis.

### Potential Pitfalls

Many factors make extrapolation from the laboratory to the clinic difficult and inaccurate. We tend to use differing endpoints for laboratory and clinical evaluation of agents [22]. Most screening tests for drug-drug or drug-radiation interactions are based on assessing relatively early endpoints. These will assess the effects of therapy on the bulk of tumour cells but may tell nothing about the effects of this therapy on the rare tumour stem cells whose death or survival will determine whether the patient enjoys long term local disease control or response followed by recurrence. *In vitro* endpoints such as dye exclusion, metabolic activity, apoptosis, and short term colony formation and *in vivo* ones of tumour regression and re-growth delay are all assays of differentiated rather than stem cells. At present only *in vivo* assays of local control truly address the issue of elimination of stem cells. We have only slowly come to realise that tumors are not composed simply of tumour cells in great numbers, but are organised tissues with stroma, vasculature, and tumour cells, and that models addressing only a single one of these components will rarely be accurate reflections of the clinical situations. The recent development and modest success of anti-vascular agents such as Bevacizumab in combination with radiation and chemotherapy attests to the wisdom of the late Judah Folkman’s insight of many years ago that targeting tumour vasculature might be worthwhile.

### Quality assurance in radiation oncology trials

The increasing precision in determining macroscopic tumour extent as well as radiation dose delivery which have come with the development and introduction of 4D multimodality imaging and IGRT have increased the need for robust quality assurance programs in clinical trials as well as routine clinical care. The assessment of a potentially radio sensitising drug will be confounded when there is substantial heterogeneity in the radiation dose delivered to patients on a clinical trial designed to evaluate its efficacy. During the past decades it has become routine for Clinical Cooperative Groups and most commercially funded trials to implement rigorous radiation therapy quality assurance (RTQA) procedures for credentialing radiation therapy facilities prior to their participating in a trial and to monitor individual cases. Even with these efforts, major variation rates of 5-10% are not uncommon, even for relatively straightforward treatments (e.g. lateral opposed fields for prophylactic cranial irradiation) and the potential for significant variation increases with more complex treatments [23-28].

### Institutional Obstacles

Pober *et al*. have reviewed the obstacles facing academic medical centres conducting translational research [29]. While their examples came primarily from the areas of vascular biology and organ transplantation, the general principles they identified apply equally well to oncology:

- Inadequate financial support
- Shortage of “translational investigators”
- Impediments in the academic culture to collaboration
- Academic Medical Centre structural organisation often hinders collaboration
- Regulatory impediments to translation
- Absence of mechanisms for facilitation of translational research

So long as collaboration is not actively encouraged and incentivised, the cultural, administrative, and practical (where to meet, can you get surgeons, medical and radiation oncologists, and biochemistry post-docs to agree on what a ‘good’ meeting time is) impediments to good translational research will make it the exception rather than the rule.

### Emerging Applications and Issues for Radiation Oncology

The following are a number of areas of current translational research in radiation oncology which exemplify some of the foregoing issues. Many others could be considered.

1. The definition of target volumes for radiation therapy is coming to rely increasingly on functional as well as the traditional anatomic information. Techniques including PET with FDG or other tracers, MR spectroscopy, and functional MRI give us the potential to image such biological parameters as proliferation rate, hypoxia, specific gene expression, and correlation of anatomy with neurologic function (e.g. language). With appropriate image fusion this will allow us to sculpt radiation doses to volumes biologically deemed appropriate for dose escalation [30]. While this is an appealing approach there are few data to indicate its practicality or correctness and clinical trials will need to validate that the imaging indeed correlates with function, that complex heterogeneously planned dose distributions are indeed delivered accurately, and capture detailed patterns of failure data to correlate with delivered dose. The implications for the RTQA process are formidable.
2. The development of hypo-fractionated RT for NSCLC, pancreatic adenocarcinoma, and other malignancies has major interplay with both physics and biology. In physics, this has involved determination of, and adaptation to, real-time tumour motion with respiration and cardiac activity, plus development of better algorithms for calculating doses with small beams and air-tumour tissue heterogeneities. In biology, the effect of large fraction size, long overall treatment time, hypoxia, and the effects of radiation on vasculature as well as tumour cells come into play. We are badly in need of better methods of assessing tumour response and local control in view of the marked fibrotic changes developing following such regimens in the chest and the frequent intercurrent death of patients who have been treated with such regimens.
3. Observations in patients with malignant mesothelioma as well as lung cancer treated with complex conformal or IMRT plans in which large volumes of lung are treated to low doses (e.g. 5Gy during the entire course of treatment) have increasingly shown that the dose-volume relationships developed for patients treated for lung cancer and Hodgkin’s disease do not adequately predict pulmonary toxicity [31]. The dose distributions outside the ‘high-dose volume’ with these newer planning approaches are very different from those from which we had previously developed ‘safe’ dose levels, which were based primarily on relatively simple APPA and oblique field arrangements. The possible role of low dose hypersensitivity in lung tissue and the interaction between radiation and concurrent chemotherapy will also have to be considered as we develop new guidelines for tolerance of thoracic irradiation [32].
4. To what degree can we accept and widely implement (i.e. pay for) new approaches based on intermediate endpoints? Or do we need to show a clear survival or quality of life benefit before adopting ‘promising but unproven’ treatments? The use of proton beams to supplement or replace photon beams in radiation oncology is an example of an emerging technology whose true clinical value (local control and complication rates) in many common malignancies may become known only after decades, but where intermediate endpoints (radiation dose distributions in phantoms) are being used to justify the large cost of construction of such facilities and where there may be substantial financial rewards for early adopters. The physical properties of proton beams differ from those of photon beams in several ways, particularly in the deposition of a large amount of energy at the end of their range (the Bragg peak) and very rapid dose falloff after this. As a result, the relative distributions of dose to target and normal tissue volumes from proton beams should in theory be superior to those from photon beams, leading to a better therapeutic ratio. While this is undoubtedly true in theory, there is some question as to whether it is actually being achieved with present implementation of proton beams in which the beam modulation is not as advanced as with photon IMRT. Concerns have also been raised about the late carcinogenic effects of neutron contamination using the current implementations of scattered proton beams. But the greater question is whether this *in silico* dosimetric superiority will translate to a clinically meaningful difference in outcomes, and whether we can move to implement this expensive technology as routine treatment for common malignancies such as prostate, lung, and breast cancers without clinical demonstration of benefit. (For paediatric malignancies there seems a stronger consensus that the ability of proton therapy to reduce radiation dose to normal tissues in young patients, for whom both growth impairment and the risk of second malignancies are major concerns with IMRT, will make proton therapy the treatment of choice.) For common adult malignancies, the Radiation Oncologic community is rather sharply divided on this question and whether randomised Phase III trials are necessary or ethical before this technology becomes widely adopted and advertised [33-42]. Unfortunately, the issue is becoming one in which the scientific concerns are being overwhelmed by both uncritical enthusiasm that protons are the ‘magic bullet’ of radiotherapy and can be easily implemented in many facilities and by critics who assume that the high costs of current proton facilities cannot come down substantially in the future [43-45]. Some have argued that the benefits of protons are as obvious as those of parachutes, another medical therapy adopted without randomised trials [46-47]. I would argue that comparing proton and photon radiotherapy is more like comparing two differing designs of parachutes, for which randomised trials might not be out of the question. There is also the perception by some that there is a lot of money to be made by early adopters, before either clinical or regulatory reality sinks in. “The interest in protons has also been fueled by the perception that, although (or, perhaps, because) proton facilities are expensive, proton therapy can be highly profitable.” [48]

**Figure 1:**
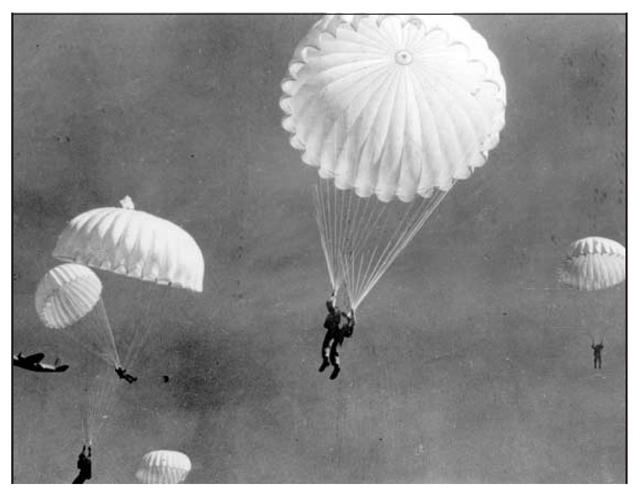
No randomised trials have shown a survival advantage for the use of parachutes when leaving airplanes at altitude. (Smith GCS, Pell JP. Parachute use to prevent death and major trauma related to gravitational challenge: systematic review of randomised controlled trials. BMJ 2003; 327: 1459-1461)

### The Roles of Government and Industry

Neither laboratory nor clinical research is done in an ivory tower or in a clinic isolated from the pressures of society. Despite rigorous scientific design and the availability of suitable patients, these forces can raise significant impediments to the execution of translational research.

The limitation of research on embryonic stem cells in the United States during the past decade is one recent example of this. Despite strong scientific arguments favouring the unrestricted establishment and clinical investigation of embryonic derived stem cell lines in many areas of medical research, Federal restrictions adopted in 2001 forbade the use of Federal research funds for stem cell research except with a limited number of lines which had already been established. Laboratories wishing to work with ‘unauthorised’ lines were required to set up redundant parallel mechanisms in the laboratory so that equipment purchased with Federal funding for approved research would not be used for work with these unauthorised lines. Faced with this additional expense, a number of programs dropped their stem cell research programs, or have had to expend additional time searching for alternative private or state funding. Numerous scientific bodies and patient advocacy groups have argued cogently but to date unsuccessfully against these limitations, which appear based more on political expediency than any reasoned scientific argument [49].

Commercial funding of clinical and translational research can also introduce serious constraints on the design of clinical trials. This is an understandable result of the differing goals of the pharmaceutical or device manufacturer, which is to bring an effective product to market and benefit its stockholders, and clinical researchers. Manufacturers will be reluctant to fund trials which run a high risk of showing their product in a less than favourable light, either ineffective or toxic, particularly before it receives approval from the FDA for its primary indication. This has often limited evaluation of new agents with potential clinical application as radiosensitisers until after their initial approval. A second problem arises when it is desired to study two or more agents which are being developed by different manufacturers. While there may be a strong scientific reason to study the combination, such as their inhibiting different components of a signaling pathway, pharmaceutical companies have been highly reluctant to make their developing agents available for such study, again largely for fear that toxicities noted for the combination may delay approval of their agent. The balance between patient protection and speedy development of promising new agents is a delicate one under the best of circumstances but is complicated further by commercial interests.

One might think that after the successful testing of a basic hypothesis in a well-conducted clinical trial, the hard work would be over and the newly validated therapy would see rapid clinical adoption. Unfortunately, this has often not been the case in radiation oncology, particularly in trials which have involved altered fractionation. Two recent examples in lung cancer are good examples of this problem.

The North American Intergroup trial 0096 compared two fractionation schemes, 45Gy/30fx/3wks (BID fractionation) and 45 Gy/25fx/5 wks (QD fractionation). The BID regimen had been designed based on laboratory observations of the minimal shoulder on the radiation dose-survival curve for SCLC cell lines and the rapid clinical growth of this disease, and had been tested in Phase II trials with encouraging results. Mature results of this trial published in the New England Journal of Medicine showed a statistically significant improvement in survival (17% vs. 27%) at five years favouring the BID regimen [50]. The BID regimen did cause more acute Grade 3 esophagitis but other toxicities were similar for the QD and BID regimens. Yet few Radiation Oncologists in the US have adopted this as a standard regimen, only about 10%. The more popular (but not validated in Phase III trials) approach has been to increase the total tumour dose to 60-65 Gy using daily fractionation. This variation between evidence-based and common practice has been recognised by leaders of the US clinical cooperative groups for more than five years during which there have been several proposals for a randomised trial comparing a higher dose QD regimen to the 45Gy BID regimen, which have been turned down by the NCI on several occasions. We thus remain in a position where an evidence-based standard has not been adopted by the practice community.

A similar situation exists in NSCLC. Again, based on laboratory data on accelerated repopulation of tumour clonogens during a protracted period of fractionated radiotherapy, investigators developed accelerated fractionation schedules in which fraction size was modestly reduced (e.g. to 1.5-1.8 Gy) but two or three fractions given per treatment day in order to shorten the overall treatment time. The prototype of such regimens was the British CHART (Continuous Hyperfractionatd Accelerated Radiation Therapy) regimen which treated patients for three fractions per day, seven days a week, delivering 54 Gy in 36 fractions over 12 treatment days. In prospective trials this was shown to be equivalent to conventional fractionation for patients with squamous cell head and neck cancer and superior to conventional fractionation for both local control and overall survival for patients with NSCLC. A subsequent trial conducted in the US compared a modification of CHART called HART, which eliminated the weekend treatments and increased total dose to 57.6 Gy in 36 fractions over 2.5 weeks, to conventional fractionation in patients with Stage III NSCLC following two cycles of induction carboplatin and paclitaxel and found an improvement in median survival of 14.9 to 20.3 months and three-year survival from 14% to 34% [51]. These differences unfortunately did not reach statistical significance because the trial was closed prematurely with only half of its planned accrual.

Macbeth surveyed radiotherapy centres in the UK two years after the publication of these results and found that only two of 22 were offering CHART to patients with lung cancer [52]. Several others were considering such implementation but had not yet done so. He proposed that three reasons were possible for this lack of adoption; the evidence was not believed, the current financial climate limited more labour intensive fractionation strategies, or that there were not enough patients who fit the entry requirements of the CHART trials in routine clinical practice to make it worthwhile. He further speculated that behind these ‘reasonable’ concerns lay three more important reasons not so likely to be articulated publically: changing workflow patterns and practice culture is difficult, particularly without strong financial or academic incentives, the potential value of curative radiation therapy for patients with NSCLC is undervalued in the oncologic community, and, since CHART required no new drugs or radiotherapy technology to purchase it is without a champion in the marketplace. The general failure of altered fractionation schemes to make major inroads into clinical practice, while not confirming his suspicions, is certainly consistent with them. We seem to prefer to radiosensitise with cisplatin than with fractionation even when the results are similar.

The development of better targeted and possibly individualised therapies raises new questions about the marketing of these agents. An agent which can be marketed as being effective, albeit only marginally so, for all patients with a common disease such as lung cancer has a huge potential market. A more selective agent with a much greater likelihood of activity but only in a select subgroup of patients with the correct pattern of molecular targets has, from the standpoint of industry, a much smaller potential market, yet similar development costs as a ‘me-too’ drug for a common indication. This leads to the realistic fear that the development and marketing of these effective but niche agents may be left to languish, and some would argue that the current status of radiolabelled antibody therapy for patients with non-Hodgkin’s lymphomas is an early example of this phenomenon (53).

## DISCUSSION

Translational research in its broader sense involves not the single step of taking a clever idea from the laboratory into clinical trial, but a coordinated series of steps back and forth between these two partners, while a large number of not-disinterested parties including society as a whole, its governmental representatives, public and private funding sources, regulatory bodies look on from the side.

Academic conflicts of interest based on pride and desire for advancement can be no less compelling and distorting of reality than the obvious financial conflicts of interest which come with stock ownership or commercial funding of research. The recent and ongoing controversy over the indirect funding of a major trial evaluating CT-based screening for lung cancer by a foundation established by the Liggett Group, a major tobacco company, illustrates that even the perception of possible distortion of research objectives and study design can undermine confidence in the results [54]. Although some have questioned whether patients are as concerned with such conflicts of interest so long as active new treatments are developed, it seems highly likely that governmental and academic regulatory agencies will be even more stringent in keeping a clear separation between the commercial funding of medical research and its design and publication. Unfortunately, with US government funding of cancer research failing to match inflation and increasing regulatory costs, clinical investigators increasingly find themselves in the painful situation of having an exciting variety of agents and combinations to test and little money to do so at a time when advances in cancer biology give real promise of more effective treatments.

## RECOMMENDATIONS

While it seems an intuitively good idea to bring together various specialists involved in the treatment of a disease, it has not always been easy to demonstrate objectively the benefits of such an approach. Several studies of lung cancer treatment before and after the organisation of multidisciplinary clinical teams have found relatively little change in either intermediate endpoints such as time between diagnosis and treatment, percent of patients receiving multimodality treatment, or more important clinical endpoints such as disease-free or overall survival. It may be the case that these were well-functioning programs before the institution of formal teams. But it is also possible that the effective implementation of multidisciplinary teams, whether among clinicians of varying specialties or the more complex admixture of clinicians and basic scientists, requires active management to be successful and cannot be left to random variation and selection (through grant funding) of effective modes of organisation. This may indeed be one, probably the only valid, application of intelligent design to biology. Yet the application of formal management skills and operations research to multidisciplinary cancer management is relatively recent.

Analysis of key milestones in the development of drugs which have successfully negotiated the process from promising to established has shown how tediously long this process has been [10]. Agents were selected as successful based on citation frequency. The average lag between publication of the first article describing the preparation, isolation, or synthesis of a new agent, and the publication of the first highly cited article on its clinical application was 24 years (interquartile range 14-44 years). A secondary analysis which considered other drugs in the same class found an even longer lag (median 27 years, interquartile range 21-50 years). The authors observed that “Successful translation is demanding and takes a lot of effort and time even under the best circumstances; making unrealistic promises for quick discoveries and cures may damage the credibility of science in the eyes of the public.” They recommend several steps to improve the present system:

- Give proper credit and incentives to high quality clinical research including that designed to evaluate earlier claims of effectiveness of agents.
- Encourage collaborations between basic and clinical scientists.
- Require large, robust randomised clinical trials as the criterion of effectiveness of promising new therapies.
- For common diseases, the research focus should be more on developing novel agents and technologies rather than demonstrating a real but minor benefit from an established therapy. It is unlikely that an established treatment with a major benefit has gone unnoticed.

An additional constraint on entry of patients to clinical trials has been the reluctance of some health insurers and other payers to extend coverage for treatments deemed experimental, whether or not these are part of a formal clinical trial. In some cases not only the experimental portion of treatment but the entire treatment course has been turned down. Attempts to better define the actual costs of participation in clinical trials through such efforts as the Cost of Cancer Treatment Study may help convince payers that trials which can properly identify effective treatments (and conversely indicate which treatments are ineffective and should not be re-imbursed) may be both scientifically valuable and cost effective [55]. The increasing willingness of carriers to support Oncotype® testing for women with breast cancer, with the understanding that the identification of low risk women who do not need adjuvant chemotherapy benefits not only the patient but saves the insurance carrier the costs of chemotherapy, may be one example of the convergence of scientific, clinical, and financial objectives.

The movement of senior investigators from university-based to industrial laboratory-based research, and back again has increased in recent years, as cultural barriers to such participation have faded, grant funding become more scarce, and federal limitations on stem cell research constrained some lines of investigation except with private funding [56]. While such increased flexibility may be all to the good it should not be forgotten that the need to produce a marketable product has the potential to distort the goals and means of commercial research just as much as the desire for publication and promotion can sully the academic research enterprise. Hopefully the incremental and ultimately self correcting methods of the scientist, whether basic or clinical, will be able to withstand such pressures in the long run.

Fortunately, there is increasing interest in the more efficient design and organisation of the basic, translational, and clinical biomedical research enterprise. This is stimulated both by frustration with the high cost and slow progress of the systems which have developed in the past as well as recognition that the revolutions in molecular biology, synthetic chemistry, medical imaging, and medical informatics both mandate and empower new approaches to clinical trial design. Considerations range from modified statistical design, use of alternative endpoints to the old 1 or 2 dimensional response criteria, particularly for agents expected to be cytostatic rather than cytotoxic, and better engineering of molecular specificity for ‘multitargeted’ agents with both activity and toxicity in model systems. This creative ferment in trial design, along with an increasingly robust understanding of tumour biology, provides the scientific underpinning for a strong future for translational research in oncology.

Finally, it must be kept in mind that we have not completed our task when we have developed an effective new therapy, but rather when we have made it available to the patients in need of it. Such translation ‘from bench to bedside to community’ remains a challenge in much of medicine and will require our personal and social commitment in the coming decades [67].
